# Editorial: Psychological dynamics in knowledge management: cognitive, emotional, and social influences

**DOI:** 10.3389/fpsyg.2026.1995346

**Published:** 2026-10-20

**Authors:** Patrick S. W. Fong

**Affiliations:** School of Engineering and Built Environment, Griffith University, Southport, QLD, Australia

**Keywords:** cognitive processes, emotions, knowledge hiding, knowledge management, knowledge sharing, organizational behavior, psychological safety, social dynamics

Knowledge management (KM) is often described through processes: creating, capturing, sharing, retaining, and applying knowledge. Yet, none of these processes is psychologically neutral. Before knowledge can be codified or transferred, it must be noticed, encoded, interpreted, trusted, voiced, and acted upon. The seven contributions to this Research Topic bring that human substrate into sharper focus. Taken together, they show that effective KM is not simply a technological or structural challenge; it is a cognitive, emotional, and social accomplishment unfolding across individuals, teams, organizations, and digital environments.

The Research Topic begins with the cognitive foundation of knowledge itself. Chen et al. review evidence on cognitive control and memory encoding, showing that control is not uniformly beneficial: task switching, interference inhibition, and response inhibition can affect what is remembered in different ways. The implication for KM is fundamental. Knowledge systems may make information available, but availability does not guarantee encoding, retention, or later retrieval. The design of knowledge-intensive work must therefore take seriously the limits and selectivity of human attention and memory.

Liu et al. extend this cognitive lens from the individual mind to the information environment. Their study of online emergencies shows how anchoring, representativeness, and availability heuristics shape cognitive bias through adaptive expectations and implicit attributions, with meaningful differences across demographic groups. In digital knowledge environments, therefore, the problem is not only whether information reaches people, but how context frames what appears credible, memorable, and actionable. KM must concern itself with the quality of sensemaking, not merely the quantity of information circulated.

From cognition, the Research Topic moves naturally to the emotional and relational conditions of knowledge sharing. Zhang and Xu show that psychological capital is connected to knowledge sharing through emotional intelligence and psychological safety among e-commerce practitioners. Their findings position hope, optimism, resilience, self-efficacy, emotional regulation, and interpersonal safety not as peripheral “soft” variables, but as resources that help determine whether knowledge is exchanged. Bai adds a leadership perspective, demonstrating that transformational leadership supports employee career growth partly through knowledge sharing, while self-efficacy and psychological capital shape the strength of these relationships. Together, these studies make a clear point: people share what they know when they feel capable, supported, and safe enough to do so.

The next contributions ask what happens when knowledge must be mobilized under pressure. Wu et al. show that pilots' personal knowledge management capability is positively associated with career resilience, with safety behavior acting as a partial bridge between knowledge capability and resilient functioning. Their non-linear findings for safety climate and work pressure are equally important: supportive conditions can strengthen adaptation, but “more” is not always “better.” Knowledge becomes valuable when it is translated into disciplined action within an appropriate psychological and operational climate.

At the team level, Yang and Suntrayuth make this translation especially visible. Drawing on knowledge creation and dynamic capability perspectives, they show that team knowledge dynamics contribute to resilience through communicative, leadership, interpretive, and analytical capacities. Knowledge creation alone is insufficient; teams must discuss, interpret, judge, and enact what they know. Resilience, in this sense, is an emergent achievement of collective cognition and coordinated action.

Wang et al. then carry the argument to the organizational level. Their study of technology-oriented small and medium-sized enterprises (SMEs) identifies a learning-resilience pathway through which strategic human resource management supports innovation. Organizational learning builds knowledge; resilience enables adaptation; together they help convert human resource practices into innovation. Yet, the inverted U-shaped role of environmental dynamism reminds us that psychological and knowledge capabilities are always contingent. When turbulence becomes excessive, established routines and capabilities may no longer be enough.

Across these seven papers, three themes converge. First, psychological dynamics are not antecedents sitting outside KM; they are part of the mechanism through which knowledge is acquired, interpreted, shared, and applied. Second, KM is best understood as a multilevel process: cognitive control and personal resources scale into interpersonal exchange, team sensemaking, resilience, learning, and innovation. Third, context matters. Task demands, leadership, psychological safety, safety climate, digital information fields, human resources (HR) systems, and environmental dynamism can enable knowledge to become action—or distort and constrain that translation. Figure 1 synthesizes these themes, illustrating how cognitive, emotional, and social influences shape knowledge management processes and adaptive outcomes across individuals, teams, and organizations within enabling or constraining contexts.

**Figure 1:**
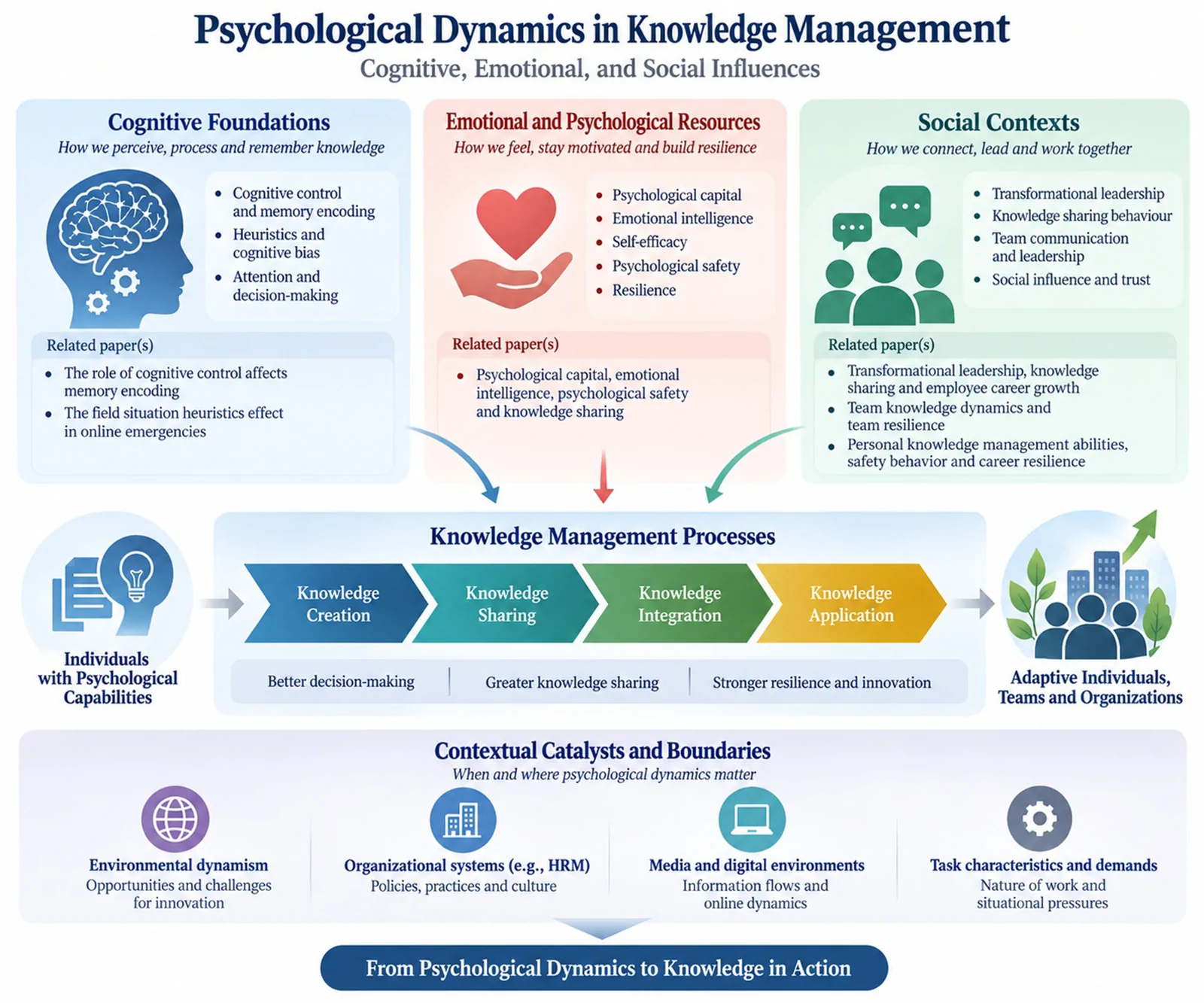
From psychological dynamics to knowledge in action: a multilevel synthesis of the Research Topic and the seven contributions. The conceptual content and framework were developed by the author, and the visual was generated with the assistance of ChatGPT (OpenAI, 2026). The figure does not present original empirical data.

The agenda opened by this Research Topic is therefore both practical and intellectually demanding. Future work should connect levels of analysis more deliberately, combine self-report evidence with longitudinal, behavioral, neurocognitive, and digital-trace methods, and examine whether psychological mechanisms travel across occupations, cultures, and technologies. This is especially important as artificial intelligence (AI) becomes embedded in knowledge work: the central question will not only be what AI can capture or generate, but how human attention, emotion, trust, agency, and social interaction shape what people accept, share, challenge, and ultimately use.

The enduring message of this Research Topic is simple: **knowledge does not manage itself**. Between information and intelligent action stands the human mind in a social world. Understanding that space is essential if KM is to become more adaptive, resilient, and genuinely useful.
